# Specificity: A Phenotypic Comparison of Communication-Relevant Domains Between Youth With Down Syndrome and Fragile X Syndrome

**DOI:** 10.3389/fgene.2018.00424

**Published:** 2018-10-01

**Authors:** Laura del Hoyo Soriano, Angela John Thurman, Leonard Abbeduto

**Affiliations:** ^1^MIND Institute, University of California, Davis, Sacramento, CA, United States; ^2^Department of Psychiatry and Behavioral Sciences, University of California, Davis, Sacramento, CA, United States

**Keywords:** Down syndrome, fragile X syndrome, phenotype, language, communication, false belief understanding, behavior

## Abstract

Despite the shared presence of an intellectual disability (ID), there is a growing literature documenting important phenotypic differences between Down syndrome (DS) and fragile X syndrome (FXS). These conclusions, however, are based on a synthesis across studies, each of which typically includes only measures of a limited number of constructs, and with differing participant characteristics. Firmer conclusions regarding specific phenotypes require a single comprehensive multi-domain assessment of participants with the syndrome groups being well matched on chronological age (CA) and cognitive functioning. The current study was designed to fill this gap by assessing several important cognitive and behavioral domains relevant to communication, such as: structural language skills, false belief understanding, as well as pragmatics and behavioral difficulties, in 30 adolescents of both sexes with DS and 39 males with FXS, matched on CA and nonverbal (NV) cognition. After statistically controlling for NV cognition, we did not find significant syndrome differences in expressive and receptive structural language or false belief understanding. In contrast, participants with DS displayed less stereotyped language and fewer behavioral difficulties compared to males with FXS. Within-syndrome associations among the targeted domains are described. Finally, females with DS were less impaired than males with DS in almost all structural language domains, whereas no significant sex-related differences were observed in NV cognition, false belief understanding, pragmatics, or behavior. Clinical and methodological implications of the findings are discussed.

## Introduction

Down syndrome (DS) is typically caused by an extra copy of all or part of chromosome 21 (Patterson, 2007). It is the most common known genetic cause of intellectual disability (ID) and, except in relatively infrequent cases of translocations, DS is not inherited (Grieco et al., 2015). Fragile X syndrome (FXS)-the leading inherited cause of ID- is caused by the expansion of a CGG trinucleodide repeat located in the *FMR1* gene on the long arm of the X chromosome (Saldarriaga et al., 2014). Despite the presence of an ID and, therefore, increased risk of communication impairment in both conditions (Abbeduto and Hagerman, 1997; Foley et al., 2015), important phenotypic differences have been observed between DS and FXS. For example, challenging behavior and pragmatic (i.e., social) language problems appear to be more common or severe in FXS, whereas structural language deficits (e.g., syntactic), although present in FXS, are greater in DS (Abbeduto and Hagerman, 1997; Chapman and Hesketh, 2000; Abbeduto and Chapman, 2005; Abbeduto et al., 2007; Finestack et al., 2009; Finestack and Abbeduto, 2010). Social-cognitive impairments, which contribute to communicative difficulties, seem to be comparable in degree across the two syndromes, although the types of errors made in social-cognitive processing may be specific to each etiology (Abbeduto et al., 2001; Cornish et al., 2005).

It is important to recognize, however, that most cross-syndrome studies have assessed only a few domains of functioning and often have not controlled for other relevant differences between the syndromes, such as cognitive ability. Moreover, participant characteristics, such as chronological age (CA) and sex, have differed across study samples. The current study was designed to fill the gaps in our knowledge of the syndrome-specific phenotypic profiles of DS and FXS, after matching/controlling for CA and nonverbal (NV) cognition, by assessing several important cognitive and behavioral domains related to communication, including structural language, social cognition, pragmatic skills and behavioral difficulties in 30 adolescents of both sexes with DS and 39 males with FXS. It is important to note that because FXS is an X-linked condition, females with FXS are, as a group, less severely affected than males (Hagerman et al., 1992). More specifically, although there is a wide range of functioning observed, depending on the proportion of active cells that have the non-affected X chromosome (Loesch et al., 2004; Ligsay and Hagerman, 2016), less than a third of females with FXS have an ID, making a comparison with females with DS (or even males with FXS) difficult to interpret. Thus, females with FXS were excluded from the present study.

Regarding language skills, there is considerable research documenting that structural language skills (i.e., syntax, vocabulary, phonology) constitute one of the most impaired aspects of the cognitive profile of individuals with DS. Indeed, expressive syntax is especially impaired, with performance lagging relative to both NV cognitive and vocabulary level-expectations (Finestack and Abbeduto, 2010; Channell et al., 2015; Lee et al., 2017). At the same time, there is evidence of sex-related variability in structural language skills in the DS population, with females displaying a longer mean length of utterance (MLU) in words, and using richer vocabulary and syntax than males from a very young age (Berglund et al., 2001). Hearing loss and differences in oral-facial anatomy and physiology also negatively impact speech perception and production in both males and females with DS (Stoel-Gammon, 2001), but with considerable within-syndrome variability in this regard (Karmiloff-Smith et al., 2016).

Males with FXS also demonstrate structural language production impairments relative to CA expectations. However, findings are somewhat mixed as to whether their skills in this domain are more delayed than expected based on level of cognitive functioning (Roberts et al., 2007a; Kover and Abbeduto, 2010; Estigarribia et al., 2011). Some studies comparing males with FXS to cognitively matched typically developing (TD) children, show expressive and receptive grammar and vocabulary consistent with mental age (MA) expectations (Abbeduto et al., 2003; Price et al., 2007; Roberts et al., 2007d; Finestack and Abbeduto, 2010). In contrast, other studies show significantly poorer structural language skills in males with FXS relative to younger TD children with similar NV MAs (Roberts et al., 2007a; Price et al., 2008; Martin et al., 2013). In addition, conductive hearing loss associated with recurrent otitis media is often observed in individuals with FXS and may contribute to problems with the structural aspects of expressive language (Barnes et al., 2009), just as in the case of DS (Stoel-Gammon, 2001).

Direct comparisons of structural language difficulties between DS and FXS have been relatively infrequent, and the findings inconsistent across studies. Some studies have not shown differences between the two conditions (Hogan-Brown et al., 2013), whereas several other studies suggest that those with FXS have better syntactic skills, both receptively and expressively, than their cognitively matched peers with DS (Price et al., 2007, 2008; Finestack and Abbeduto, 2010).

Regarding social cognition, most of the studies performed in individuals with FXS or DS have generally focused on impairments in their Theory of Mind (ToM)—the “theory” that other people have intentions, thoughts, beliefs and emotions, and that these various epistemic states influence people's behavior and how they interact with others, as well as an understanding that other people's epistemic states can differ from one's own (Carpendale et al., 2007). With regard to DS, there is paucity of research on ToM, perhaps reflecting the assumption that children with DS have relatively strong social skills. However, recent studies have shown that children and adolescents with DS exhibit problems relative to CA expectations with higher-order cognitive processing of social information affecting interpersonal understanding (Fidler, 2006; Cebula et al., 2010).

In the case of those with FXS, it has been well documented that males with FXS are impaired relative to CA expectations with regard to ToM, having difficulties in differentiating appearance from reality (Cornish et al., 2005). In addition, males with co-morbid FXS and autism spectrum disorder (ASD) have greater deficits than those with FXS alone (Lewis et al., 2006); however, it has been suggested that both subgroups of FXS are superior as regards ToM performance to that of individuals with idiopathic ASD (Abbeduto and Murphy, 2004).

A cross-syndrome comparison study of males with DS and males with FXS (matched on verbal MA) (Cornish et al., 2005) showed comparable difficulties between the groups in the ability to understand beliefs and intentions, although qualitative differences in error types between the groups were observed. Realist errors, ignoring the appearance of an object and instead relying solely on real knowledge, were more common in FXS, whereas participants with DS tended to demonstrate more phenomenist errors, relying on perceptual information about an object even if it contradicts real knowledge of that object. In another study, it was found that a group of males with FXS, ranging in age from childhood to young adulthood, performed better on a false belief task assessing ToM than did a group of individuals with DS who were CA- and NV MA-matched (Abbeduto et al., 2001). Note that differences in language skill did not fully explain the group differences in this latter study. In addition, some studies comparing DS and FXS to TD have reported asynchrony between false belief understanding and cognitive level for adolescents with DS, but not for adolescents with FXS (Abbeduto et al., 2001; Losh et al., 2012). However, when comparing FXS to other IDs of unknown etiology, a similar asynchrony has been found in males with FXS (Grant et al., 2007). Thus, findings about whether the understanding of false beliefs is on par with cognitive level expectations are mixed for both DS and FXS.

Regarding pragmatics, or the use of language to achieve social ends, there is considerable evidence of performance below cognitive level-expectations in males with FXS. In addition to delays in acquiring basic pragmatic skills (Abbeduto et al., 2007), males with FXS also display atypical pragmatic behaviors. Most notable in this regard is perseveration, or the excessive repetition of a word, phrase, or topic in conversation (Abbeduto and Hagerman, 1997). Perseveration is more common in FXS than in either males with DS or TD of similar MA (Abbeduto and Hagerman, 1997). Males with FXS also tend to produce more off-topic and tangential language than individuals with DS (Wolf-Schein et al., 1987; Sudhalter et al., 1990; Roberts et al., 2007c). In addition, differences in narrative, or storytelling, skills, such as fewer story actions used in their narrative retellings, are observed for males with FXS relative to MA-matched TD individuals (Estigarribia et al., 2011).

In contrast, youth with DS are generally thought to have a relative strength in pragmatic skills, at least in those skills reflecting social motivation. Indeed, a recent set of studies conducted with children and adolescents with DS suggests relatively intact nonverbal communication skills (Smith et al., 2017), and rates of parent-reported social relationships and frequencies of attempts to repair conversational breakdown that are similar to TD MA-matched controls (Johnston and Stansfield, 1997; Laws and Bishop, 2004). At the same time, however, other aspects of pragmatics are observed to be quite impaired in those with DS, including requesting (Coggins et al., 1983; Beeghly et al., 1990), topic initiation and elaboration (Tannock, 1988; Roberts et al., 2007c), appropriately maintaining a topic of conversation (Tannock, 1988; Beeghly et al., 1990), providing clear referential descriptions (Abbeduto et al., 2006), recognizing and requesting clarification of unclear messages (Abbeduto et al., 2008), use of stereotyped language (Laws and Bishop, 2004), and understanding the contexts of conversation (Smith et al., 2017), with performance below MA-expectations in several of these areas. In addition, sex-related differences in pragmatic abilities in favor of females have been found in DS, although the extent of these differences varies as a function of CA, overall cognition, and methods of assessment (e.g., semi-naturalistic conversational context vs. standardized methods) (Lee et al., 2017).

When comparing pragmatic abilities in DS and FXS, several studies suggest that individuals with DS have fewer difficulties than males with FXS, at least with respect to the behavioral and social-motivational components of pragmatic skills (Wolf-Schein et al., 1987; Sudhalter and Belser, 2001; Roberts et al., 2007b). It's unclear, however, the extent to which the higher rates of anxiety and ASD symptomatology observed in FXS relative to DS (Moss et al., 2013; Thurman et al., 2014; Niu et al., 2017)—which are further detailed in the section below—contribute to these patterns of between-group differences in pragmatics. However, males with FXS have been found to have more complete knowledge of appropriate language for diverse social situations than individuals with DS (Martin et al., 2013). Therefore, specific pragmatic deficits linked to each syndrome may exist and are likely related to other aspects of their cognitive, behavioral and social phenotypes (Martin et al., 2009; Estigarribia et al., 2012).

Regarding behavioral problems, individuals with FXS, especially males, generally display a variety of impairments that emerge early in childhood (Rivera and Reiss, 2009). These include hyperactivity, hyperarousal, and unusual or exaggerated responses to sensory stimuli, impulsivity, and inattention (Turk, 1998; Munir et al., 2000; Cornish et al., 2007), aggression and self-injury (Symons et al., 2003), social anxiety (Merenstein et al., 1996; Cordeiro et al., 2011), and ASD-like behaviors (Kau et al., 2004; Clifford et al., 2007). Those ASD-like behaviors present in more than 90% of males with FXS (Feinstein and Reiss, 1998; Bailey et al., 2000) include perseverative and noncontingent speech (Martin et al., 2012), motor stereotypies such as hand flapping, and poor eye contact (Merenstein et al., 1996; Roberts et al., 2007b; Oakes et al., 2016).

In contrast, individuals with DS have been claimed to display fewer behavioral problems than individuals with other ID conditions, including FXS (Tonge and Einfeld, 2003; Foley et al., 2015). Nonetheless, some aspects of behavior are impaired compared to the general population (Næss et al., 2017). For example, individuals with DS have trouble controlling impulses and managing frustration in late childhood and adolescence (Merrick et al., 2004). This frustration may be related to the barriers they experience due to the difficulties expressing themselves or understanding others (Chapman and Hesketh, 2000; Shapiro and Accardo, 2010). As individuals get older, those externalizing behaviors tend to decline, whereas increased internalizing symptoms (e.g., social withdrawal, depression, anxiety, secretive behavior) are more likely to occur (Grieco et al., 2015). Finally, within DS, there is some, albeit inconsistent evidence, of sex differences with regard to behavioral difficulties; indeed, some studies have suggested increased behavior problems in males (Van Gameren-Oosterom et al., 2011, 2013), whereas others have reported increased problems in females (Dykens et al., 2002), and still others have found no differences (Jacola et al., 2014).

The inconsistency of the results across investigations targeting cognitive and behavioral skills may be due to several differences among studies, including differences in the CA and sex of the participants, the specific skills examined (e.g., different aspects of social-cognition, such as false belief understanding, joint attention, facial recognition), the methods used (e.g., naturalistic tasks vs. standardized tasks, directly assessed vs. caregiver-reported), the units of measure (e.g., growth scores, raw scores, scaled scores), the context of assessment (e.g., conversation vs. narration or clinic vs. home), the domain or method by which groups are matched (e.g., matched on NV MA vs. matched on verbal MA) (Phillips et al., 2014). Virtually every study described in the foregoing review focused on measuring a single or limited set of skills or behavioral difficulties in a relatively narrow domain of functioning. Moreover, relatively few of these studies directly compared appropriately matched groups of participants with DS or FXS specifically; instead, usually comparing a sample of participants with a single syndrome to a matched TD comparison group. Although such studies are useful, stronger conclusions regarding phenotypic features specific to a syndrome requires a comprehensive multi-domain assessment of participants and with comparison of the two syndrome groups matched, ideally, on CA and level of cognitive functioning or degree of intellectual disability. Matching on an indicator of overall cognitive level provides a benchmark against which skills likely to depend on cognitive ability, such as structural language, can be judged as relatively strong or weak across the syndromes (Abbeduto et al., 2016). Matching on CA is useful for providing a benchmark against which to judge skills and behaviors, such as adaptive behavior and even challenging behavior, that are highly dependent on generalized experience in the world and linked to cultural demands (e.g., entry into, or exit from, school) as well as behaviors judged to be inappropriate because they violate CA expectations, such as fear of separation from a parent (Boer, 2006; Scattone et al., 2011). That said, matching youth with DS and males with FXS on both (CA and cognitive level) is difficult, mainly because males with FXS have, on average, higher IQs than same-CA youths with DS (Finestack et al., 2013; Kover et al., 2013; Channell et al., 2014). Thus, matching on both parameters may require a combination of equating through participant selection and statistical adjustment.

The current study was designed as to provide a comprehensive comparative approach to examine syndrome-related phenotypes by assessing several communication-related domains—structural language, social cognition, pragmatics and behavioral difficulties—in a single integrated assessment of participants with DS and FXS. The groups were matched on CA through participant selection and on NV cognition through statistical adjustment. This approach made it possible to determine the syndrome specificity regarding the assessed communication-related domains (1) by identifying differences and similarities between the two syndromes as regards level of functioning in each domain. (2) We also examined within-syndrome interrelationships between structural language skills, social cognition, pragmatic abilities, and behavioral problems in each etiologic group. In addition, (3) we examined differences between males and females with DS in the domains of skill and behavior we assessed and the relationships among these domains.

## Methods

### Participants

Sixty-nine individuals between the ages of 10 and 16 years with intellectual disability (NVIQ ≤ 70 ± 5) were included in the current study. We included 39 males with FXS and 30 participants with DS (20 males and 10 females). Because FXS is X-linked and females with the full mutation are far less impaired than males, with less than one-third meeting criteria for an intellectual disability, females with FXS were excluded from the current study. Participants were screened to ensure that (a) they used speech as the primary mode of communication, (b) regularly used three-word or longer phrases, (c) were native English speakers, and (d) had no major uncorrected physical or sensory impairments that would interfere with the ability to perform the tasks included in the research protocol. Hearing problems were directly assessed in both FXS and DS groups by establishing pure tone thresholds and requiring a threshold of 30 dB or better in one ear. Parents gave their written informed consent before their son or daughter participated. Descriptive demographic and clinical data are presented in Table 1. The present data are derived from the first annual assessment of the longitudinal study. The participants in the present study were part of a larger longitudinal study, which has generated several previous publications. Several of the language measures included in the present analyses have been included in other published studies from the larger longitudinal project (Pierpont et al., 2011; Kover et al., 2012; McDuffie et al., 2012; Finestack et al., 2013) however, none of these previous studies included the entire battery of language, pragmatic, social-cognitive, and behavioral measures included in the present study, and previous studies only focused on selected subsets of the cohort reported on here (e.g., only FXS males, only the most verbally capable individuals, or only individuals without a comorbid diagnosis of autism spectrum disorder).

**Table 1:** Sociodemographic characteristics and clinical parameters.

**Down syndrome (*n* = 30)**	**Fragile X syndrome (*n* = 39)**
MEAN [SD (range)]	
**Age**	
12.7	12.8
(1.8[10–16])	(1.8[10–16])
**Leiter-R IQ**	
42.7	46.8
[7(36–65)]	[10(36–73)]
**Leiter-R GS**	
462	466.9
[7.4 (442–474)]	[10 (446–490)]
**Sex**	
20 males 10 females	39 males
**Bilingual**	
1	1
**Race**	
Caucasian 29	Caucasian 35
African American 0	African American 1
Native 0	Native 1
Asian 0	Asian 0
Hispanic 1	Hispanic 1
Other 0	Other 1
**Genetics**	
Full trisomy 25	Full mutation 21
Partial trisomy 0	Premutation 0
Translocation 0	Mosaic 12
Mosaic 0	Cytogenetic 4
Unknown 5	Unknown 2

*SD, standard deviation; n, number of participants; IQ, intellectual quotient; GS, growth score*.

### Procedures and measures

Participants completed a test battery that was administered over the course of two (typically consecutive) days. In most cases, the entire battery was administered to any given participant by the same examiner at any given annual assessment. All measures were administered by well-trained graduate-level research assistants with extensive experience working with individuals with developmental disabilities. The measures presented in this paper are only a subset of those comprised in the battery, concretely those targeting domains relevant to communication skills.

#### Direct assessments

##### Nonverbal cognition

The Brief IQ subtests of the Leiter International Performance Scale—Revised (Leiter-R; Roid and Miller, 1997) were administered. The Leiter-R is a nonverbally administered (i.e., pantomimed) assessment in which no verbal responses are required. The subtests comprising the Brief IQ are: Figure Ground, Form Completion, Sequential Order, and Repeated Patterns, with the former two domains reflecting skills in the visualization domain and the latter two reflecting skill in the domain of fluid reasoning. Growth Scores (GS) were used as the metric of NV cognitive functioning because of the floor effects observed for IQ (standard scores) for the DS group (28.6% of the DS sample obtained the minimum score of 36 and exhibited a significant absolute skewness index (>2). GS are equal-interval scores, also known as derived rasch scores, which are conceptually similar to age-equivalents and reflective of absolute ability (as opposed to IQ, which is age referenced) and have a mean of approximately 500 for fifth graders.

##### Structural language assessments

Four standardized measures were administered. Receptive vocabulary was assessed using the Peabody Picture Vocabulary Test, Third Edition (PPVT-III; Dunn and Dunn, 1997). In this task, the participant selects the drawing from a four-drawing array that best matches the meaning of a word spoken by the examiner. The raw score (i.e., total number of correct answers) was used in the present analyses. The Test for Reception of Grammar, Second Edition (TROG-2; Bishop, 2003a) was administered to evaluate receptive syntax. The TROG-2 assesses comprehension of phrases and sentences of varying syntactic complexity, with items focused on word order, function words, and grammatical inflections. Participants select the one picture from an array of four that matches the meaning of the utterance spoken by the examiner. Items are presented in blocks of four, with all items in the block exemplifying the same syntactic construct (e.g., the plural morpheme). The total number of blocks passed (i.e., all four items answered correctly) was used in the present analyses.

Expressive language was assessed using the Expressive Vocabulary Test (EVT; Williams, 1997) and the Syntax Construction Test from the Comprehensive Assessment of Spoken Language (CASL; Carrow-Woolfolk, 1999). In the EVT, expressive vocabulary knowledge is assessed using pictures and examiner prompts to solicit labels or synonyms. The CASL Syntax Construction (CASL-SC) subtest requires generating sentences embodying a variety of targeted morphosyntactic rules. Sentences are elicited by asking the participant to formulate a word, phrase, or sentence that is semantically and grammatically compatible with a verbal stimulus and drawing. For both measures, we used raw scores (i.e., the number of items answered correctly) to avoid the floor effects observed in the standard scores.

Expressive language abilities were also assessed in a conversational context following procedures developed by Abbeduto and colleagues (Abbeduto et al., 1995; Kover et al., 2012; Berry-Kravis et al., 2013). These procedures involve a set of topics to be introduced as well as scripts for introducing and following up on the topics, with the goal being to minimize examiner talk, encourage participant talk, and obtain 10 min of conversation. Conversations were audio-recorded and later transcribed and analyzed using the Systematic Analysis of Language Transcripts (SALT; Miller and Chapman, 1999). Five dependent measures were derived with the unit of analysis being the C-unit (i.e., an independent clause and its modifiers, which could include dependent clauses): *talkativeness* (number of C-units attempted per minute), *unintelligibility* (proportion of C-units that were partly or completely unintelligible), *dysfluency* (proportion of C-units that contained a verbal dysfluency, false start, or filler), *lexical diversit*y (number of different words in 50 complete and intelligible units or the full sample if less than 50 C-units), and *syntactic complexity* (mean length of utterance in morphemes, or MLU). See Kover et al. (2012) for details of the transcription process.

##### Social cognition

We administered a slightly modified version of the false belief task (FBT) developed by (Benson et al., 1993), which was designed to assess aspects of ToM. In the task, two stories are read aloud and enacted with props. Each story concerns a character who believes that a desired object is hidden in one location, but the participant knows that the object is actually hidden in another location. At the conclusion of each story, the participant is asked two test questions to determine whether he or she can discriminate between his or her true belief about the object's location from the story character's false belief about the object's location. In addition to the test questions, control questions are asked, some of which are designed to determine whether the participant has processed, and can recall, critical events in each story, and some of which are structurally similar to the test questions, but contain no mental state language, so as to determine whether the participant can manage the linguistic structure of the test questions. The two stories differed in that one required reasoning about a character's beliefs about the world (first-order reasoning), whereas the other required reasoning about a character's beliefs about yet another character's beliefs about the world (second-order reasoning). The order of presentation of the two stories was random across participants. Our modifications of the Benson et al. task consisted of minor wording changes in the story and control questions, as well as increased standardization of the story enactment.

#### Caregiver report measures

Biological mothers served as informants for all caregiver report measures.

Pragmatic skills were assessed through the Children's Communication Checklist (CCC-2), (Bishop, 2003b). The CCC-2 is a 70-item questionnaire designed to obtain information about children's communication abilities and behaviors. In the present study, we only included data from the four subscales focused on pragmatic abilities (inappropriate initiation, stereotyped language, use of context, and NV communication). We used scaled scores for the present analyses, with a low score indicative of a more severe pragmatic impairment.

Behavioral problems were assessed through the Child Behavior Checklist, Ages 6–18 (CBCL/6–18) (Achenbach and Rescorla, 2001). The CBCL/6–18 is comprised of 118 items regarding children's competencies and behavioral/emotional problems. Caregivers rate their child for how true each item is now or within the past 6 months using the following scale: 0 = not true (as far as you know); 1 = somewhat or sometimes true; 2 = very true or often true. A number of different scales and metrics are generated form the CBCL/6–18. We focused only on those scales reflecting especially challenging areas for one or both of the syndromes of interest: *Anxious/Depressed; Attention Problems; Social Problems; Thought Problems;* and *Withdrawn/Depressed*. We used T scores. Higher scores reflected greater problems.

### Statistical analysis

Descriptive statistics were computed for all variables. Cognitive, language, social-cognitive, and behavioral variables from direct assessments in which more than 10% of the sample obtained the maximum or the minimum score, and/or exhibited a significant absolute skewness index (>2) were categorized as having ceiling or floor effects.

We first compared the DS group (including both males and females) to the CA-matched group of males with FXS on each of the dependent variables. In order to quantify the magnitude of the differences between DS and FXS, Cohen's effect size (“Cohen's *d”*), which is the difference of the means of two independent samples divided by the pooled standard deviation was calculated, along with the 95% confidence interval (Cohen, 1988). Because standardized Cohen's *d* do not depend on the nature of the scores, they are particularly useful in cases in which different measures of the same constructs are scored using different scales not directly comparable (Dunst and Hamby, 2012). We categorized each difference as *large* (effect size differences larger than 1 pooled standard deviation (|*d*| > 1), *substanti*al (|*d*| > 0.7), *medium* (|*d*| > 0.5), or *small* (|*d*| > 0.3). Less than 0.3 pooled standard deviation was considered as *not meaningfully different*. In these comparisons, we designated FXS as the standard, which means that positive values in Cohen's *d* correspond to higher scores in the DS group, whereas negative values in Cohen's *d* represent lower DS scores in comparison to the FXS group. Note that Leiter-R, FBT, PPVT, TROG, EVT, CASL-SC, ELS-*Talkativeness*, ELS-*Lexical Diversity, and* ELS-*Syntactic Complexity*, as well as all subscales from CCC-2 assess skills, whereas ELS-*Dysfluency*, ELS-*Unintelligibility*, as well as all subscales from CBCL/6-18 assess difficulties.

We next conducted an ANOVA, comparing DS and FXS, with separate analyses for each dependent variable, in order to adjust the ANOVAs *p*-values for multiple comparisons (method described below). Differences were considered to be statistically significant if the resulting *p*-values were less than 0.05 after controlling for multiple comparisons. Because the groups were not matched on Leiter-R GS [Cohen's *d* −0.55 [−1.04, −0.05], *p-*value < 0.5, (Kover and Atwoo, 2013)], we next examined group differences on the cognitive and behavioral variables after controlling for differences in Leiter-R GS using an ANCOVA analysis. We included syndrome (DS and FXS) as a between-participants factor and Leiter-R GS as a covariate. Differences were considered to be statistically significant if the resulting *p*-value was less than 0.05 after controlling for conducting multiple ANCOVAs.

We also compared males and females within the sample of participants with DS. Here we first relied on Cohen's *d* method to categorize the differences between males and females in terms of the magnitude of the effect sizes. We next conducted an ANOVA, comparing males and females with DS, with separate analyses for each dependent variable, in order to adjust the ANOVA *p*-values for multiple comparisons (described below). Group differences were considered to be statistically significant if the resulting *p*-value was less than 0.05 after controlling for conducting multiple ANOVAs. Because males and females with DS were matched on age and NV cognition, there was no need to include either as a covariate. Therefore, ANCOVAs were not applied for the within-DS analyses.

To explore the within-syndrome associations among NV cognition, structural language skills, social-cognitive abilities, pragmatic skills, and behavioral problems, correlation coefficients were calculated separately for each syndrome group. Pearson or Spearman coefficients were used depending on results for each variable in the Shapiro-Wilk test of normality. When appropriate, partial correlations were computed to control for Leiter-R GS. In these analyses, correlations were considered to be statistically significant if the resulting *p*-value was less than 0.05 after controlling for conducting multiple within-group correlations (described below).

Because a large number of statistical comparisons were made, we applied the False Discovery Rate (FDR) procedure (Benjamini and Hochberg, 1995) in each set of analyses in order to control for multiple comparisons. This procedure essentially controls for multiple comparisons by specifying the proportion of significant results that could be false positives in contrast to a procedure such as the Bonferroni correction that controls for the number of tests conducted within a family of tests. It should be noted that the FDR approach is less conservative than is the Bonferroni approach. Note that the FDR-adjusted *p*-values are presented as *q*-values in tables and text if remaining significant. In addition, we have reported only moderate and strong correlations (*r* ≥ 0.4) that were significant after controlling for multiple comparisons.

All statistical analyses were performed using the statistical software packages SPSS (Version 18.0; SPSS Inc., Chicago, IL, USA) and R (Version 3.1.1; The R Foundation for Statistical Computing, Vienna, Austria).

## Results

### Descriptive demographic and clinical data of the participants

Socio-demographic data and clinical descriptors of the DS and FXS participants are provided in Table 1.

### Cognitive, language, and behavioral performance in DS participants compared to FXS males

Descriptive statistics, Cohen's *d*, and confidence intervals (95% CI) for the comparison of participants with DS to those CA-matched males with FXS on the dependent variables are summarized in Table 2. Regarding structural language, the Cohen's *d* indicated that there were s*ubstantial differences* for the PPVT and CASL, and *medium differences* were observed for the EVT, TROG, lexical diversity and syntactic complexity in conversation, all with greater deficits in DS than FXS. *No significant differences* between groups were suggested by the Cohen's *d* in the remaining conversation measures (i.e., talkativeness, unintelligibility, and dysfluency).

**Table 2:** Descriptive analyses, Cohen's *d*, and confidence intervals (95% CI) and ANCOVA models adjusted for NV cognition on dependent measures for males and females with DS compared to CA-matched males with FXS.

**Groups:**	**Down syndrome (males and females)**			**Fragile X syndrome (males)**			**Cohen's-d ^a^ (*p*-value, *q*-value)**	**95% CI**	**Adjusted for NV cognition**			
	**Mean (SD)**	**Range (min-max)**	***n***	**Mean (SD)**	**Range (min-max)**	***n***						
Chronological Age	12.7 (1.81)	10–16	30	12.8 (1.78)	10–16	39	0.089	[−0.4, 0.57]		**Covariate-adjusted Mean**		
**NV Cognition**									***p*****-value (*****q*****-value)**	**F**	**DS**	**FXS**
Leiter-R GS	461.96 (7.4)	442–474	29^1^	466.89 (9.97)	446–490	37^2^	−**0.55 (0.030, 0.046)**	**[**−**1.04**,−**0.05]**				
**Social Cognition**												
False belief understanding (FBT)	0.14 (0.18)	0–0.75	29^1^	0.25 (0.27)	0–1	37^2^	−0.48 (0.06, –)	[−0.97, 0.02]	0.66	0.2	0.20^b^	0.23^b^
**Receptive Structural Language**												
Receptive grammar (TROG)	2.4 (1.63)	0–6	30	4.07 (3.67)	0–15	38^1^	−**0.57 (0.023, 0.045)**	[−1.05, −0.07]	0.59	0.29	3.37^b^	3.70^b^
Receptive vocabulary (PPVT)	64.93 (23.06)	8–107	30	86.13 (31.12)	55–156	38^1^	−**0.76 (0.003, 0.012)**	[−1.25, −0.26]	0.10	2.89	76.09^b^	85.04^b^
**Expressive Structural Language**												
Expressive vocabulary (EVT)	44.7 (13.5)	0–74	29^1^	56.49 (19.48)	34–103	37^2^	−**0.69 (0.007, 0.02)**	**[**−**1.18**, −**0.18]**	0.23	1.5	51.08^b^	55.05^b^
Syntax construction (CASL-SC)	6.31 (6.39)	0–26	29^1^	13.48 (9.98)	0–36	35^4^	−**0.84 (0.001, 0.005)**	**[**−**1.34**, −**0.32]**	0.10	2.79	9.34^b^	12.45^b^
Talkativeness (ELS)	12.47 (3.46)	7–19.9	27^3^	12.77 (3.52)	6.5–20.20	34^5^	−0.09 (0.75, –)	[−0.59, 0.42]	0.44	0.61	12.44^b^	11.66^b^
Unintelligibility (ELS)	16 (11.84)	0–57	27^3^	11.97 (8.84)	0–24.26	34^5^	0.39 (0.14, –)	[−0.12, 0.99]	0.31	1.06	15.81^b^	12.46^b^
Dysfluency (ELS)	22.73 (16.5)	0.95–66	27^3^	20.10 (12.74)	1.54–44.62	34^5^	0.18 (0.48, –)	[−0.33, 0.69]	0.05 (—)	3.9	25.10^b^	16.80^b^
Lexical diversity (ELS)	72.29 (29.15)	0–123	27^3^	89.85 (30.57)	59–157	34^5^	−**0.59 (0.027, 0.045)**	**[**−**1.09**,−**0.06]**	0.52	0.42	77.22^b^	82.80^b^
Syntactic complexity (ELS)	3.42 (1.18)	1.84–5.89	27^3^	4.22 (1.49)	2.45–8.24	34^5^	−**0.59 (0.026, 0.045)**	**[**−**1.10**, −**0.07]**	0.69	0.16	3.71^b^	3.85^b^
**Pragmatic Skills (CCC-2)**												
Less inappropriate initiation	5.27 (2.13)	2–9	26^4^	3.94 (2.21)	0–8	33^6^	**0.61(0.023, 0.045)**	**[0.08, 1.13]**	0.21	1.61	5.10^b^	4.26^b^
Less stereotyped language	5.12 (2.26)	1–12	26^4^	3.33 (2.33)	0–7	33^6^	**0.77 (0.005, 0.017)**	**[0.23, 1.30]**	<**0.01 (0.02)**	**8.19**	**5.32**^b^	**3.29**^b^
Proper use of context	1.27 (1.51)	0–6	26^4^	1.82 (2.19)	0–8	33^6^	−0.29 (0.3, –)	[−0.80, 0.23]	0.25	1.35	1.30^b^	1.99^b^
NV communication	3.81 (1.83)	1– 8	26^4^	2.73 (1.51)	0–5	33^6^	**0.65(0.016, 0.04)**	[0.12, 1.17]	0.05 (—)	3.8	3.77^b^	2.87^b^
**Behavioral Problems (CBCL/6-18)**												
Withdrawn behaviors	58.73 (6.54)	50–73	30	62.58 (8.07)	50–79	38^1^	−0.52 (0.038, –)	[−1.00, −0.02]	<**0.01 (0.01)**	**10.1**	**56.54**^b^	**63.27**^b^
Anxious symptoms	52.93 (4.67)	50–67	30	60.89 (8.22)	50–83	38^1^	**-1.16 (**<**0.001**,<**0.001)**	**[**−**1.66**, −**0.63]**	<**0.001(**<**0.001)**	**19.19**	**52.29**^b^	**60.31**^b^
Social problems	59.56 (4.56)	50–69	30	63 (7.68)	51–83	38^1^	−**0.53 (0.034, 0.049)**	**[**−**1.01**, −**0.04]**	0.19	1.75	59.71^b^	62.31^b^
Thought problems	60.1 (7.42)	50–75	30	66.65 (8.07)	50–77	38^1^	−**0.84 (0.001, 0.005)**	**[**−**1.33**, −**0.33]**	<**0.01 (0.006)**	**12.20**	**58.69**^b^	**66.52**^b^
Attention problems	58.06 (5.01)	51–73	30	65.65 (8.5)	50–92	38^1^	−**1.06 (**<**0.001**,<**0.001)**	**[**−**1.56**, −**0.54]**	<**0.01 (0.006)**	**11.94**	**57.95**^b^	**65.90**^b^

NV, nonverbal; SD, standard deviation; n, number of participants; d, differences; CI, confidence interval; F, Fisher-Snedecor quotient; GS, growth score; FBT, False Belief Task; TROG, Test for Reception of Grammar; PPVT, Peabody Picture Vocabulary Test; EVT, Expressive Vocabulary Test; CASL-SC, Syntax Construction Test from the Comprehensive Assessment of Spoken Language; ELS Expressive Language Sample; CCC-2, Children's Communication Checklist, CBCL, Child Behavior Checklist;

1 *1 missing value*,

2 2 missing values„

3 *3 missing values*,

4 *missing value*,

5 5 missing values„

6 6 missing values

a *Cohen's effect size. Large differences (effect size differences larger than 1 pooled standard deviation (|d| > 1); Substantial differences (|d| > 0.7); Medium differences (|d| > 0.5); Mild/small differences (|d| > 0.3)*,

b *Covariates appearing in the model are evaluated at Leiter-R GS = 465.12 value (the mean). Note: q-values represent p-values after controlling for multiple comparisons according to FDR procedure, we only reported significant q-values, otherwise this symbol (—) is included as a sign of not passing the FDR criterion. Significant results after controlling for multiple comparisons according to FDR procedure are bolded in the table*.

With respect to social cognition, Cohen's *d* suggested that there were *no meaningful differences* in the FBT. In reference to the Cohen's *d* for the pragmatic domains of the CCC-2, s*ubstantial differences* were found on stereotyped language and *medium differences* for both NV communication and inappropriate initiation, with greater parent-reported skill for DS than FXS. Finally, Cohen's *d* indicated that there was *no significant difference* between groups for proper use of context on the CCC-2.

Regarding the Cohen's *d* analyses for the CBCL/6-18, *large differences* were observed for anxiety and attentional problems, *substantial differences* for thought problems, and *medium differences* for withdrawn-depressed symptoms and social problems. In all cases, parents reported lesser concerns for DS than FXS.

The results for the ANCOVA in which the DS participants were compared to the males with FXS are also reported in Table 2. After controlling for Leiter-R GS, there were no significant differences between DS and FXS on any of the structural language variables, NV communication on the CCC-2, or and social problems on CBCL/6-18. Interestingly, inclusion of Leiter-R GS as covariate had no effect on the group differences in stereotyped language on the CCC-2 or on withdrawn behaviors estimated means, anxious symptoms, or thought and attention problems (CBCL/6-18)—all of those differences were significant following the application of the FDR (see Table 2).

### Impact of sex in DS participants

Descriptive statistics, Cohen's *d*, and confidence intervals (95% CI) for the comparison of females with DS and males with DS—matched on both CA and NV cognition—on each of the dependent variables are presented in Table 3. Cohen's *d* revealed *large differences* (*d* > 1) on nearly all measures of structural language, with all differences reflecting better performance on the part of the females with DS. The exceptions were talkativeness and dysfluency in conversation, which were found to fall in the *no significant differences* category according to Cohen's *d*. We also found that the Cohen's *d* suggested that there were *no significant differences* between males and females with DS on any of the measures of social cognition, pragmatic skills, or behavioral problems.

**Table 3:** Descriptive analyses, Cohen's *d*, and confidence intervals (95% CI) for cognitive, linguistic, social-cognitive, and behavioral performance of males with DS compared to females with DS matched on CA and NV cognition.

**Groups:**	**Down syndrome (males)**			**Down syndrome (females)**			**Cohen's-d^a^ (*p*-value, *q*-value)**	**95% CI**
	**Mean (SD)**	**Range (min-max)**	***n***	**Mean (SD)**	**Range (min-max)**	***n***		
Chronological Age	12.7(1.98)	10–16	20	12.7 (1.5)	10–14	10	0.01	[−0.78, 0.79]
**NV Cognition**								
Leiter-R GS	460.32 (7.20)	442–474	19^1^	465.1 (7.11)	450–474	10	−0.66 (0.1, –)	[−0.14, 1.43]
**Social Cognition**								
False belief understanding (FBT)	0.11 (0.14)	0–0.5	19^1^	0.2 (0.22)	0–0.75	10	−0.51 (0.3, –)	[−1.27, 0.28]
**Receptive Structural Language**								
Receptive grammar (TROG)	2.05 (1.57)	0–6	20	3.10 (1.6)	1–5	10	−0.66 (0.1, –)	[−1.42, 0.13]
Receptive vocabulary (PPVT)	59.40 (24.07)	8–98	20	76 (16.93)	43–107	10	−0.75 (0.06, –)	[−1.51, 0.49]
**Expressive Structural Language**								
Expressive vocabulary (EVT)	40.11 (12.97)	0–72	19^1^	53.4 (10.1)	42–74	10	−**1.10 (0.009, 0.034)**	**[**−**1.88**, −**0.26]**
Syntax construction (CASL-SC)	3.79 (3.44)	0–16	19^1^	11.1 (8.03)	1–26	10	−**1.35 (0.002, 0.013)**	**[**−**2.15**, −**0.47]**
Talkativeness (ELS)	12.95 (3.57)	6.9–17.9	17^3^	11.66 (3.3)	7.5–17.6	10	0.37 (0.4, –)	[−0.43, 1.15]
Unintelligibility (ELS)	20.44 (11.99)	8.57–57	17^3^	8.42 (6.97)	0–22.67	10	**1.15 (0.008, 0.034)**	**[0.28, 1.95]**
Dysfluency (ELS)	21.27 (17.87)	0.95–66	17^3^	25.23 (14.43)	1.12–53.71	10	−0.24 (0.6, –)	[−1.01, 0.55]
Lexical diversity (ELS)	60.06 (21.91)	0–90	17^3^	93.1 (28.93)	36–123	10	−**1.34 (0.002, 0.013)**	**[**−**2.15**, −**0.44]**
Syntactic complexity (ELS)	2.87 (0.77)	1.84-4.32	17^3^	4.35 (1.2)	2.34–5.89	10	−**1.55 (0.001, 0.013)**	**[**−**2.39**, −**0.63]**
**Pragmatic Skills (CCC-2)**								
Less inappropriate initiation	5.53 (1.97)	2–8	17^3^	4.78 (2.44)	2–9	9^1^	0.35 (0.4, –)	[−0.47, 1.53]
Less stereotyped language	5.24 (2.41)	1–22	17^3^	4.89 (2.09)	3–8	9^1^	0.15 (0.7, –)	[−0.66, 0.96]
Proper use of context	1.35 (1.54)	0–6	17^3^	1.11 (1.54)	0–4	9^1^	0.16 (0.5, –)	[−0.66, 0.96]
NV communication	4 (2.12)	0–8	17^3^	3.44 (1.13)	2–5	9^1^	0.30 (0.2, –)	[−0.52, 1.1]
**Behavioral Problems** (CBCL)								
Withdrawn behaviors	59.9 (7.12)	50–73	20	56.4 (4.67)	50–63	10	0.54 (0.2,–)	[−0.24, 1.3]
Anxious symptoms	53.8 (5.36)	50–67	20	51.2 (2.15)	50–57	10	0.57 (0.2, –)	[−0.22, 1.32]
Social problems	59.4 (4.73)	50–69	20	59.9 (4.43)	54–67	10	−0.11(0.8, –)	[−0.86, 0.65]
Thought problems	59.7 (7.95)	50–75	20	60.9 (6.52)	51–73	10	−0.16 (0.7, –)	[−0.92, 0.6]
Attention problems	57.7 (4.37)	51–64	20	58.8 (6.3)	52–73	10	−0.22 (0.6, –)	[−0.97, 0.55]

NV, nonverbal; SD, standard deviation, n, number of participants; d, differences; CI, confidence interval; GS, growth score; FBT, False Belief Task; TROG, Test for Reception of Grammar; PPVT, Peabody Picture Vocabulary Test; EVT, Expressive Vocabulary Test; CASL-SC, Syntax Construction Test from the Comprehensive Assessment of Spoken Language; ELS Expressive Language Sample; CCC-2, Children's Communication Checklist; CBCL, Child Behavior Checklist;

1 *1 missing value*,

3 *3 missing values*;

a *Cohen's effect size. Large differences (effect size differences larger than 1 pooled standard deviation (|d| > 1); Substantial differences (|d| > 0.7); Medium differences (|d| > 0.5); Mild/small differences (|d| > 0.3). Note: q-values represent p-values after controlling for multiple comparisons according to FDR procedure. We only reported those q-values that were significant after controlling for FDR, otherwise this symbol (—) is included as a sign of not passing the FDR test. Significant results after controlling by FDR procedure are bolded in the table*.

### Intercorrelations among constructs in DS participants and FXS males

We examined the associations between NV cognition (Leiter-R GS) and the remaining variables (see Table 4), doing so separately for DS participants and FXS males. Results showed that Leiter-R GS was correlated with receptive and expressive grammar (TROG and CASL), as well as with receptive and expressive vocabulary (PPVT and EVT), in both syndromes. In addition, NV cognition was associated with false belief understanding (FBT) and the conversational measures of dysfluency, syntactic complexity, and lexical diversity in the FXS group.

**Table 4:** Correlations between NV cognition and the assessed domains in DS participants and in FXS males.

	**Pearson correlations:** ***r*** **(*****p*****-value**, ***q*****-value) [95% CI]**	
	**DS group (males and females)**	**FXS group (males)**
	**NV cognition (Leiter-R GS)**	
**Social Cognition**		
False belief understanding (FBT)	**—**	**0.61 (**<**0.001, 0.001) [0.356, 0.78]**
**Receptive Structural Language**		
Receptive grammar (TROG)	**0.54 (0.002, 0.008) [0.216, 0.757]**	**0.79(**<**0.001, 0.001) [0.626, 0.887]**
Receptive vocabulary (PPVT)	**0.66 (**<**0.001, 0.001) [0.387, 0.827]**	**0.75 (**<**0.001, 0.001) [0.563, 0.864]**
**Expressive Structural Language**		
Expressive vocabulary (EVT)	**0.71 (**<**0.001, 0.001) [0.458, 0.856]**	**0.78 (**<**0.001, 0.001) [0.61, 0.881]**
Syntax construction (CASL-SC)	**0.55 (0.003, 0.011) [0.223, 0.766]**	**0.79 (**<**0.001, 0.001) [0.620, 0.889]**
Talkativeness (ELS)	**—**	**—**
Unintelligibility (ELS)	**—**	**—**
Dysfluency (ELS)	**—**	**0.42 (0.013, 0.04) [0.095, 0.664]**
Lexical diversity (ELS)	**—**	**0.55 (0.001, 0.004) [0.26, 0.749]**
Syntactic complexity (ELS)	0.45 (0.02, **—**)	**0.62 (**<**0.001, 0.004) [0.357, 0.792]**
**Pragmatic Skills (CCC-2)**		
Less inappropriate initiation	**—**	**—**
Less stereotyped language	0.42 (0.04, **—**)	**—**
Proper use of context	**—**	**—**
NV communication	**—**	**—**
**Behavioral Problems (CBCL)**		
Withdrawn behaviors	**—**	**—**
Anxious symptoms	**—**	**—**
Social problems	**—**	**—**
Thought problems	**—**	**—**
Attention problems	**—**	**—**

*NV, nonverbal; GS, growth score; FBT, False Belief Task; TROG, Test for Reception of Grammar; PPVT, Peabody Picture Vocabulary Test; EVT, Expressive Vocabulary Test; CASL-SC, Syntax Construction Test from the Comprehensive Assessment of Spoken Language; ELS Expressive Language Sample; CCC-2, Children's Communication Checklist; CBCL, Child Behavior Checklist; CI, confidence interval; r, Pearson correlation; p-value, two tailed significance; Q-value; represent p-values after controlling for multiple comparisons according to FDR procedure. Only are reported significant correlations, otherwise this symbol — is included as a sign of p > 0.05. Only are reported significant q-values, otherwise this symbol (—) is included as a sign of not passing the FDR test. Significant results after controlling for multiple comparisons according to FDR procedure are bolded in the table*.

We also examined the associations between structural language, pragmatic skills, false belief understanding and behavioral problems, doing so separately for each group. As regards the FXS group, results showed significant correlations between false belief understanding (FBT) and the following measures of structural language: expressive and receptive vocabulary (EVT, PPVT) [(*r* = 0.62, *p* < 0.001, *q* = 0.005, *CI* = 0.37, 0.786), (*r* = 0.60, *p* < 0.001, *q* = 0.005, *CI* = 0.343, 0.774)], and expressive and receptive grammar (TROG, CASL) [(*r* = 0.71, *p* < 0.001, *q* = 0.005, *CI* = 0.501, 0.841), (*r* = 0.63, *p* < 0.001, *q* = 0.005, *CI* = 0.376, 0.796)]; however, none of these correlations was significant after partialling out NV cognition. No significant intercorrelations were found between the remaining measures of structural language, false belief understanding, pragmatics and behavior (either before or after partialling out NV cognition) for the FXS group. As regards the DS group, results showed significant correlations between talkativeness and nonverbal communication (*r* = 0.65, *p* < 0.001, *q* = 0.035, *CI* = 0.351, 0.829), as well as between dysfluency and stereotyped language (*r* = 0.62, *p* = 0.001, *q* = 0.035, *CI* = 0.306, 0.812); however, none of these associations were significant after partialling out NV cognition.

## Discussion

This study was designed to determine which communication-relevant skills and behaviors, including structural language, false belief understanding, pragmatics, and behavioral difficulties, distinguish youth with DS from males with FXS and which are shared by both conditions in terms of a similar degree of impairment. We also examined the intercorrelations among domains separately for each syndrome group. In addition, sex-related differences and similarities within youth with DS were explored.

### Structural language and false belief understanding differences

Our results indicated that the poorer structural language skills of individuals with DS relative to those with FXS are likely a consequence of their lower level of NV cognition. Or put differently, when statistically matched on NV cognition, the two groups are equally impaired in terms of virtually all structural language variables examined.

The present findings regarding structural language contrast with the findings of several previous investigations that have suggested a weakness in expressive syntax for participants with DS relative to participants with FXS, even when the groups are matched on cognitive functioning (Price et al., 2007, 2008; Finestack and Abbeduto, 2010). Because the present project utilized multiple measures of structural language, including syntax measured in a standardized test and in a structured naturalistic conversation and with similar results across measures, the difference between our findings relative to other studies are not likely to be due to task or context (Kover et al., 2012; Martin et al., 2013). Instead, it is more likely that the difference between our findings and those of previous studies is due to differences in participant characteristics, such as age, sex distributions, or language criteria for enrollment.

More generally, our results make the point that conclusions about the syndrome-specific profile of DS and FXS regarding targeted structural language skills will broadly depend on the group-matching variables and covariates included in the models. At the same time, it is important to exercise caution when interpreting the results of any matching strategy. In particular, the fact that covarying NV cognition eliminates significant differences on structural language does not mean that the genetic anomalies of the syndromes are somehow not important in producing these deficits. Instead, the interpretation is that those genetic anomalies affect structural language at least partly through pervasive influences on cognition. In addition, future investigations should include other potential matching factors (e.g., verbal IQ, social anxiety, among others). Such matching could further clarify the pathways through which the genetic anomalies lead to the linguistic phenotypes of the syndromes.

Finally, we found no significant group differences in absolute level of performance on the false belief understanding task (before and after controlling for NV cognition). This is consistent with findings from previous studies (e.g., Cornish et al., 2005). Future studies may include multiple tools of assessment targeting different aspects of false belief understanding by conducting more in-depth qualitative analyses of errors as well as examining other facets of ToM (e.g., recognizing emotions in other people's facial expressions) in order to better describe each phenotype.

#### Pragmatic skills differences

As perceived by caregivers, participants with DS were found to demonstrate significantly greater competence in pragmatic skills than participants with FXS (e.g., better NV communication, less inappropriate imitations in conversations, as well as less stereotyped language) before controlling for NV cognition. However, the only group-difference that remained significant after controlling for NV cognition was stereotyped language. It may be that these differences are enhanced by other unique aspects of the behavioral and social phenotype of each syndrome. For example, relative strengths in social engagement, social orientation (Fidler et al., 2008), and prosocial responses (Kasari et al., 2003) might enable individuals with DS to learn and use NV skills to achieve communicative ends despite their limitations in structural language. In contrast, individuals with FXS, although wiling to engage in social interaction, display high rates of sensory disorders, social anxiety, poor eye contact, as well as other behavioral problems (Abbeduto and Chapman, 2005; Saldarriaga et al., 2014), all of which may limit aspects of pragmatic development and, perhaps, even lead them to atypical patterns of communication, including frequent use of stereotyped and repetitive language. Thus, the present results suggest that the pragmatic impairments associated with FXS are not simply a consequence of their cognitive delay, but also are reflective of their behavioral difficulties and difficulties with social interaction.

#### Behavioral differences

The results of the current study are consistent with previous studies in showing more severe behavioral difficulties in males with FXS than in individuals with DS. Before and after controlling for NV cognition, males with FXS displayed more behavioral symptoms reflecting problems with anxiety, withdrawal, disordered thought, and attention than the participants with DS. Thus, as was true for stereotyped language, these behavioral challenges associated with FXS appear to be syndrome specific, at least in their degree. Such findings suggest that the pathway from the *FMR1* mutation to certain behavioral and pragmatic challenges is not solely through cognition.

#### Cognitive and behavioral differences between males and females with down syndrome

Females with DS demonstrated an advantage relative to males with DS (matched on age and level of ID) with respect to all measures of expressive structural language (except talkativeness and dysfluency). These findings are consistent with other studies documenting a female advantage in language for DS (Berglund et al., 2001; Dionne et al., 2003; de Sola et al., 2015). It is important to note, however, that our study shows significant sex-related differences only in structural expressive language; no significant sex-related differences were found in receptive language, social cognition, pragmatics, or behavior. The existing literature has been mixed with regard to these latter domains. Although several studies have pointed to sex-differences in pragmatics, behavior, and communication skills (Lund, 1988; Berglund et al., 2001; Määttä et al., 2006; Van Gameren-Oosterom et al., 2011; de Sola et al., 2015; Lee et al., 2017), studies focused on the adolescent period specifically have generally shown no sex differences with regard to pragmatics (Martin et al., 2017) or behavior (Jacola et al., 2014). Taken together, these results suggest that sex differences in DS may vary as a function of chronological age and developmental level and, perhaps, the context of assessment. However, confirming this hypothesis will require use of a longitudinal approach.

### Within-syndrome intercorrelations among constructs

When examining intercorrelations among the different constructs within each syndrome, we found that NV cognition was related to standardized outcomes of receptive and expressive grammar and receptive and expressive vocabulary in both DS and FXS groups. NV cognition was also associated with conversational measures of dysfluency, lexical diversity and syntactic complexity, as well as with greater false belief understanding (social cognition), in the FXS group. These results suggest that NV cognition may contribute to expressive and receptive structural language skills in both syndromes. Indeed, no intercorrelations among the structural language, pragmatic skills, social cognitive, and behavioral measures were significant after controlling for NV cognition, further reinforcing the importance of the latter domain for both DS and FXS.

## Conclusion

A cognitive and behavioral profile of strengths and weaknesses regarding communication skills emerged for the two groups in this study. The inclusion of NV cognition as a covariate showed that genetic differences across groups lead to several significant differences in expressive and receptive language and that were closely related to their cognitive impairments. However, significant syndrome-related differences in stereotyped language and challenging behaviors favoring DS were irrespective of overall cognitive level. In addition, within the DS group, females demonstrated richer structural language skills than males, whereas no significant differences were shown in NV cognition, social cognition, pragmatics or behavior. More generally, our findings can be used to develop intervention plans, targeting areas of greatest impairments and exploiting differential associations between problem areas. For example, improved communication in DS, may require focusing on structural language, whereas improved communication in FXS may require targeting behavioral difficulties as well as structural language. At the same time it is important to note the importance of considering the type of assessment (i.e., standardized or naturalistic) in which language is elicited for individuals with intellectual disability. Our results suggest, consistent with previous investigations (Kover et al., 2012), that each context of assessment requires concrete yet overlapping language demands, having implications for conclusions drawn about language profiles and therefore targets selected for intervention plans.

Finally, we want to point that our findings may not generalize to other age periods. Indeed, the cognitive and behavioral pattern described for DS in the current study might differ dramatically at a later age as a consequence of their high risk of developing the neuropathology associated wi9th Alzheimer's Disease and the resulting dementia-like symptoms (Lott, 2012). Therefore, life-span longitudinal studies comparing DS and FXS are needed.

## Limitations and future directions

Some limitations relevant to the interpretation of our findings exist. First, the statistical approach conducted when analyzing patterns of correlations across variables within each diagnostic group did not allow us to compare groups in this regard since we did not test the differences in correlations. In other words: the fact that one correlation is significant and that another not, does not necessary mean that the correlations are statistically different from each other. However, it still provides interesting information within each diagnosis group and generates hypotheses for future research. Second, the small sample size of each diagnostic group may contribute to low statistical power to detect links between domains. In addition, it should be noted that the FDR approach used is less conservative than the Bonferroni approach. Further, the results of this study are based on the comparison between syndromes at a single point in time. Longitudinal studies are needed to understand syndrome-specific developmental patterns of structural language, pragmatics and behavior. This might help identify developmental periods that are most optimal for intervention for each condition. Finally, females with FXS were excluded from our analyses, which did not allow us to preserve sex-matching when comparing phenotypes. As in most previous studies, we excluded females with FXS because of the relatively lower severity of impairment compared to males. However, it is also true that females with FXS show a set of communication limitations (Sterling and Abbeduto, 2012) related to their extreme shyness and social anxiety. In addition, their attentional deficits further complicate their social interaction, being distracted, or forgetting pertinent information during a conversation. We are cognizant of this limitation and plan to include females with FXS in future studies. In summary, although the current study give us interesting information about the cognitive and behavioral profile of school aged children with DS and FXS, further longitudinal studies with larger samples are needed to confirm our results.

## Ethics statement

This study was carried out in accordance with the recommendations of the UC Davis Clinical Committee B. The protocol was approved by the Institutional Review Board of the University of California, Davis. Since all participants were minors with intellectual disability, parents of subjects gave written informed consent in accordance with the Declaration of Helsinki.

## Author contributions

LdH conceived and designed the analytical plan for this study, analyzed the data, interpreted the results, and wrote the manuscript. AT was responsible for constructing and validating all data sets, verifying the written description of the methods, and assisting with interpretation of the statistical findings. LA conceived the overall design of the larger study and protocol from which the data for this study were drawn, oversaw data collection, and contributed to the analytical plan for this study. All authors reviewed, edited, and approved the manuscript.

### Conflict of interest statement

The authors declare that the research was conducted in the absence of any commercial or financial relationships that could be construed as a potential conflict of interest.
